# Performance of Tai Chi Chuan and NF-κB-driven proinflammatory gene expression (IL-6, IL-1β, TNF-α) in adult chronic disease patients: a systematic review and meta-analysis of randomized controlled trials

**DOI:** 10.3389/fimmu.2026.1702830

**Published:** 2026-05-05

**Authors:** Si-Qi Zhu, Hong Wang

**Affiliations:** 1 Graduate School of Wuhan Institute of Physical Education, Wuhan, China; 2 School of Graduate, Wuhan Sports University, Wuhan, China; 3 School of Wushu, Wuhan Sports University, Wuhan, China; 4 Wuhan Institute of Physical Education and Wushu, Wuhan, China

**Keywords:** Tai Ji, NF-k B, IL-6 (interleukin 6), TNF-a (tumor necrosis factor a), IL-1β

## Abstract

**Objective:**

This systematic review and meta-analysis aims to evaluate the effects of Tai Chi Chuan on the expression of pro-inflammatory genes IL-6, IL-1β, and TNF-α—downstream of the NF-κB pathway—in adults with chronic diseases. It further explores potential anti-inflammatory mechanisms and identifies research gaps in the literature regarding these mechanisms.

**Methods:**

This study searched seven electronic databases for relevant literature, with language restrictions limited to English and Chinese. The risk of bias in all included trials was assessed using the Cochrane Risk of Bias Assessment Tool (version 2.0) and the GRADE (Grading of Recommendations, Assessment, Development and Evaluation) system. Standardized mean differences (SMD) with 95% confidence intervals (CI) were used to evaluate pooled effect sizes. P values < 0.05 were considered statistically significant. Subgroup analyses were conducted according to disease systems.

**Results:**

We retrieved a total of 1,110 relevant studies, with 20 studies ultimately included in the analysis. These covered diseases across multiple systems, including oncology, endocrinology, respiratory, and neurological disorders. To more directly reflect intervention effects, we extracted the mean ± standard deviation of change values post-intervention compared to baseline as the analysis data. After assessing publication bias and minimizing heterogeneity effects, we found that Tai Chi Chuan significantly reduced the expression of downstream target genes (SMD = -0.48, 95% CI: -0.76 to -0.19, p < 0.01), significantly down-regulated IL-6 (SMD = -0.66, 95% CI: -1.27 to -0.06, p = 0.03), and IL-1β (SMD = -0.59, 95% CI: -0.95 to -0.23, p < 0.01). while TNF-α showed a downward trend but without statistical significance (SMD = -0.28, 95% CI: -0.59 to 0.02, p = 0.07). Subgroup analysis revealed that patients with endocrine and respiratory system diseases derived the most significant benefit.

**Conclusion:**

Tai Chi can alleviate systemic inflammation in patients with chronic diseases by suppressing NF-κB-driven pro-inflammatory gene expression, demonstrating both safety and feasibility. Furthermore, we identified gaps in existing research on Tai Chi and NF-κB, particularly the lack of randomized controlled trials (RCTs). Future studies should conduct RCTs with NF-κB core proteins and factors as direct outcome measures to directly elucidate Tai Chi’s regulatory effects on the NF-κB pathway.

**Systematic review registration:**

https://www.crd.york.ac.uk/PROSPERO/, identifier CRD420251112908.

## 1 Introduction

Chronic inflammation is a prolonged, persistent inflammatory state that typically lasts for months or even years, consistently posing a major threat to the physical and mental health of patients with various chronic diseases. In the treatment of chronic conditions such as cardiovascular disease, chronic respiratory diseases, and diabetes, chronic inflammation often lacks the obvious symptoms of acute inflammation yet exerts significant impacts on the body. This contributes to substantial economic burdens and prolonged treatment cycles for chronic diseases, and is also a key factor in their high prevalence (1). At the core of chronic inflammation lies the prolonged activation of the body’s immune system. Sustained activation of both innate and adaptive immunity leads to continuous secretion of TNF-α and IL-6, causing vascular endothelial damage and promoting atherosclerosis (2).The long-term elevation of pro-inflammatory cytokines drives the development of multiple chronic diseases.

Within the molecular mechanisms of chronic inflammation, NF-κB signaling is regarded as a key pathway mediating inflammation, implicated in diverse human diseases including cancer, inflammatory conditions, cardiovascular disorders, and metabolic diseases (3).In the canonical NF-κB pathway, IL-6, IL-1β, and TNF-α form a positive feedback loop with NF-κB, sustaining continuous immune cell activation and the persistent release of pro-inflammatory factors (4).IL-1β and TNF-α, as upstream signals, activate the TNFR pathway and IL-1R pathway, respectively, activating IKK and phosphorylated IKKβ. This leads to IκB degradation, exposing the NLS of the p50/RelA dimer, which rapidly translocates to the nucleus. There, it binds to the κB site to initiate target gene transcription. Concurrently, downstream target genes IL-6, IL-1β, and TNF-α undergo transcriptional induction and are secreted (5).

In recent years, exercise intervention studies have demonstrated that prolonged physical activity not only reduces glucocorticoid sensitivity and mRNA levels of the GR gene but also causes decreased mRNA expression of NF-κB pathway-related genes (6).Long-term regular aerobic training can down-regulate the expression of multiple pro-inflammatory genes in peripheral blood mononuclear cells, including tumor necrosis factor-α and interleukin-6 (7). As a form of aerobic exercise, Tai Chi Chuan possesses the unique advantage of being a ‘mind-body exercise’. It not only integrates meditation with physical movement but also exhibits high adherence during practice, addressing the mind, body, nervous system, and immune system simultaneously (8, 9). Its low cost, convenience, and ease of learning have made it a growing choice for postoperative interventions and rehabilitation. The role of Tai Chi Chuan in alleviating inflammation among cancer survivors and chronic disease patients is increasingly recognized (10–12)°However, existing Tai Chi Chuan RCTs predominantly focus on pain (13), cognitive function (14–16), anxiety and depression (17)and isolated cytokine markers (18),lacking integrated research on Tai Chi Chuan’s impact on the NF-κB-driven pro-inflammatory gene axis.

Therefore, this systematic review aims to elucidate the effects of Tai Chi on the expression of the NF-κB-driven pro-inflammatory gene axis (IL-6, IL-1β, TNF-α) in chronic inflammation among adult patients. It seeks to reveal the intervention mechanism of Tai Chi at the level of gene expression and explore the differential effects of Tai Chi on chronic diseases across different systems.

## 2 Subjects and methods

### 2.1 Research design

This study protocol has been registered on the PROSPERO platform (Registration Number: CRD420251112908, URL: https://www.crd.york.ac.uk/PROSPERO/). The information presented herein is fully consistent with the content provided at registration. Throughout implementation, we strictly adhered to the Preferred Reporting Items for Systematic Reviews and Meta-Analyses (PRISMA) guidelines and referenced PRISMA recommendations for the fields of exercise, rehabilitation, sports medicine, and exercise science for both research and reporting (19).

### 2.2 Inclusion and exclusion criteria of the study

Inclusion criteria are as follows: Randomized controlled trials (RCTs) and eligible quasi-experimental studies must include at least two groups: a Tai Chi Chuan (TCC) intervention group and a control group. Participants are adult patients with any type, severity, or duration of disease. No restrictions are imposed on gender, ethnicity, or other demographic characteristics to ensure broad and unrestricted participant representation, thereby enhancing the generalizability of study findings. The intervention period lasted at least 8 weeks. The control group received non-surgical treatments, including but not limited to placebo controls, cognitive behavioral therapy, psychosocial support therapy, health education, standard care, or other routine treatments. The study must report measurements of at least one pro-inflammatory gene (IL-6, TNF-α, IL-1β). Multiple methods were employed to detect these pro-inflammatory factors, including multiplex magnetic bead immunoassays, flow cytometry, and enzyme-linked immunosorbent assays (ELISA) (20).The study language is restricted to English and Chinese.

The following studies were excluded (1): reviews, conference reports, commentaries, and cross-sectional studies; (2) duplicated publications; (3) studies with incomplete original data or those unable to extract outcome measures; (4) studies lacking a control group; (5) studies for which full-text access was unavailable; (6) studies involving surgical interventions; (7) animal experiments. Two reviewers screened titles and abstracts, then assessed eligibility after reading full texts.

### 2.3 Search strategy

Researchers searched seven databases (PubMed, Web of Science, OVID, EBSCO, Cochrane Library, Medline and Embase), covering all literature from January 2000 to July 2025. We conducted a combined search using the subject terms “Tai Ji”, “Interleukin-1beta”, “Tumor Necrosis Factor-alpha” and “Interleukin-6” along with their free-text variants (e.g., “Tai Chi Chuan”, “IL-1 beta”, “TNF-α” and “IL-6” etc.). Additional references were identified through manual searches of relevant literature.

### 2.4 Study selection and data extraction

One researcher independently extracted data, while a second researcher reviewed the findings. Any discrepancies were resolved through discussion and analysis. To effectively manage the included literature, we utilized EndNote software (version X9) and excluded duplicate studies. Two researchers independently screened titles and abstracts. Studies with unclear relevance were further evaluated by reading the full text. Any discrepancies during screening were resolved through discussion between the two researchers. Excluded studies were documented with reasons for exclusion.

From the included studies, researchers extracted multiple key data points, including: first author’s name, publication year, mean patient age, sample size, type of control intervention, and intervention design. Data extraction strictly adhered to predefined study grouping criteria. For studies lacking key information or critical data, the research team excluded them from subsequent in-depth analysis to ensure the accuracy and reliability of the research.

### 2.5 Quality assessment methods

To fully assess the risk of bias in the included trials, two reviewers independently applied the Cochrane Collaboration’s Risk of Bias Assessment Tool Version 2 (RoB 2) to evaluate six domains: randomization process, deviation from the intended intervention, missing outcome data, outcome measurement, selection of reported outcomes, and overall bias (21).Two non-randomized controlled trials were assessed for study quality using the GRADE evidence grading system (22). Disagreements arising during the assessment were resolved through discussion and analysis.

### 2.6 Statistical analysis

All statistical analyses were performed using STATA 17.0 software. Data for the same outcome measures across included studies were extracted and standardized to a uniform unit of pg/mL. To reflect the net intervention effect of TCC on pro-inflammatory gene factors, the mean and standard deviation of post-intervention changes relative to baseline were extracted as analytical data. This study employed the standardized mean differences(SMD) as the effect size measure. A negative SMD value indicates a more significant reduction in inflammatory biomarkers in the TCC group compared to the control group (non-TCC intervention or placebo control). SMD calculation was based on Cohen’s d, derived from the mean difference between groups (TCC vs. control) divided by the pooled standard deviation. Heterogeneity among studies was assessed using the I² statistic (23). Given observed heterogeneity, a random-effects model was employed for analysis. Sensitivity analyses were conducted for all outcome measures to evaluate result robustness, and reasons for result discrepancies were examined. We employed funnel plots combined with Begg’s test and Egger’s test to assess publication bias (24, 25).

For data reported as medians and quartiles, we first evaluated the distribution characteristics before applying natural logarithm transformation (ln(x)). The resulting mean and standard deviation were used for subsequent meta-analysis (26, 27).

## 3 Results

### 3.1 Study selection and characteristics

**Figure 1:**
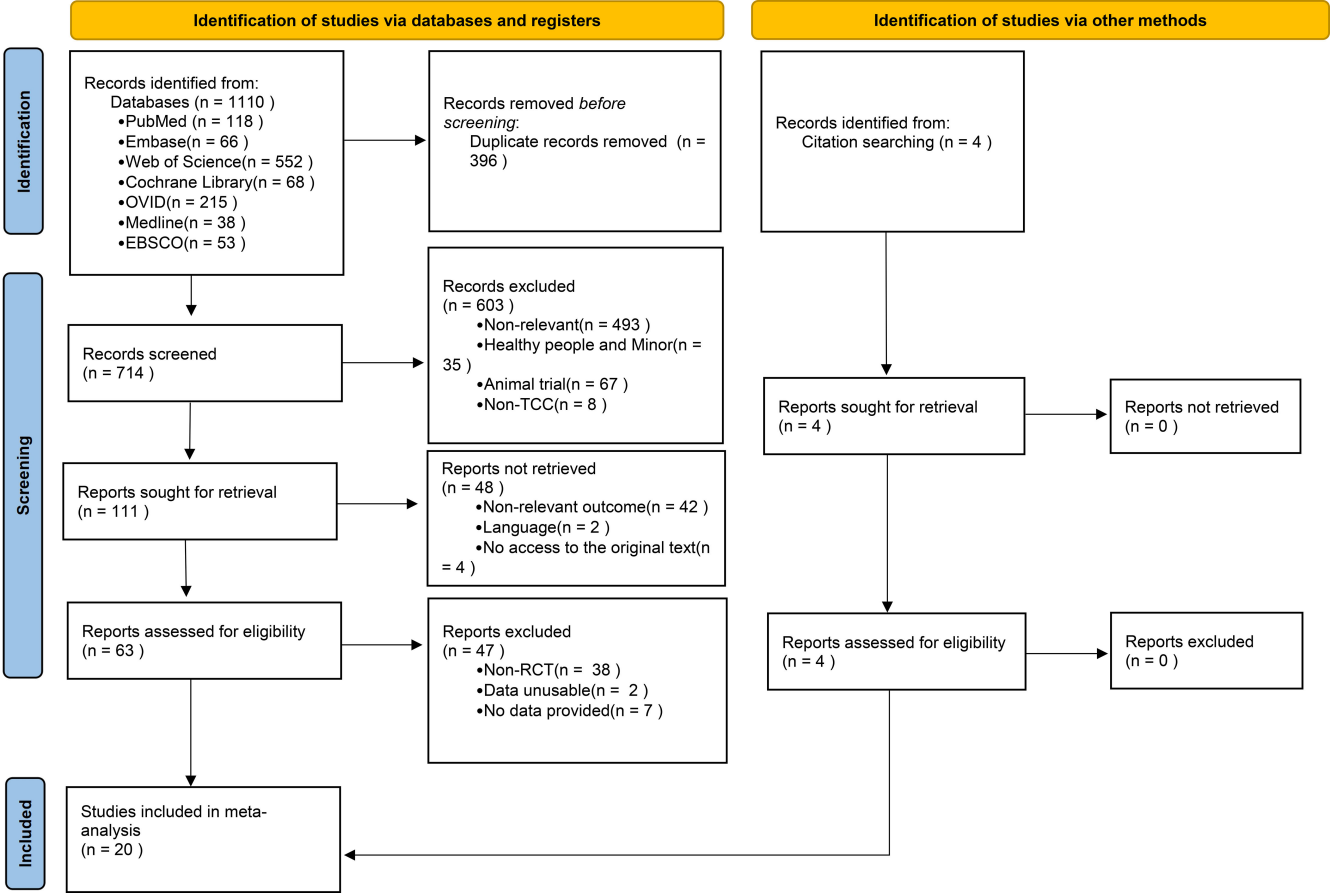
Flow diagram of the study selection process.

A total of 1,110 studies were identified and screened through literature searches. Following in-depth assessment of abstracts and titles, 63 articles were eligible for evaluation. After excluding non-randomized controlled trials, studies with unavailable data, and those lacking essential information, 16 studies were ultimately included in the meta-analysis. Additionally, four studies were identified through relevant reference lists and incorporated into the analysis (**Figure 1**).

Among the 20 included studies, patients included cancer survivors (28–32), individuals with diabetes (33–37), those with Parkinson’s disease (38) and others. Interventions in the control groups primarily comprised health education, educational control (39, 40), psychosocial support therapy, cognitive behavioral therapy, conventional therapy (37, 41, 42) and placebo controls (38, 43–45). Detailed information on other characteristics is presented in **Table 1**.

**Table 1:** Characteristics of included studies.

Author/Year	Study population	Sample size	Outcome measure	Measurement method	Intervention design	Control intervention
Campo2015	Cancer Survivors Age ≥55y	TCC=29 CG=25	IL-6, TNF-α	Multiplex Cytokine Assay Immunoassay	12 weeks, 1 hour/session, 3 times/week	HE
Cui2024	Congenital Heart Disease Patients Age 61.97 ± 9.31	TCC=14 CG=20	TNF-α	ELISA	12 weeks, 1 hour/session, 3 times/week	CERP
Du2014	Chronic Obstructive Pulmonary Disease Patients Age 64.85 ± 7.48	TCC=36 CG=38	IL-6, TNF-α	ELISA	12 weeks, 1 hour/session, twice a week	NI
Hu2022	Cancer Survivors Age 62.13 ± 6.98	TCC=19 CG=19	IL-1β	ELISA	12 weeks, 80 minutes/session, 4 sessions/week	NI
Irwin2024	Diabetes Patients Age 59.80 ± 8.63	TCC=45 CG=45	IL-6, TNF-α	MSD MULTI-SPOT	3 months, 2 hours/session, once a week	CBT
Janelsins2011	Breast cancer survivors Age 53.48 ± 8.71	TCC=9 CG=10	IL-6	ELISA	12 weeks, 1 hour/session, 3 times/week	PST
Jiang2020*	Breast cancer survivors Age 58.43 ± 9.45	TCC=50 CG=50	IL-6, TNF-α, IL-1β	ELISA	12 weeks, 1 hour/session, 7 sessions/week	UC
Li2022	Lung cancer patients Age 62.30 ± 6.18	TCC=32 CG=32	IL-6, TNF-α, IL-1β	ELISA	24 weeks, 1 hour/session, twice a week	NI
Liu2024	Parkinson’s disease patients Age 60.95 ± 5.30	TCC=16 CG=16	IL-6, TNF-α	ELISA	24 weeks, 1 hour/session, 6 sessions/week	CT
Mendoza2018	Type 2 diabetes patients Age 67.75 ± 5.62	TCC=48 CG=37	IL-6, TNF-α	Flow Cytometry	24 weeks, 50 minutes/session, 5 times/week	NI
Mendoza2014	Metabolic syndrome patients Age ≥ 60y	TCC=24 CG=25	IL-6, TNF-α, IL-1β	Flow Cytometry	24 weeks, 1 hour/session, 5 sessions/week	NI
Redwine2020	Periodontal disease patients Age 65.00 ± 8.00	TCC=18 CG=18	IL-6, TNF-α	ELISA	16 weeks, 1 hour/session, twice weekly	CT
Sprod2012	Heart failure patients Age 53.48 ± 2.91	TCC=9 CG=10	IL-6	ELISA	12 weeks, 1 hour/session, 3 times/week	SST
Sungkarat2018	Breast cancer survivors Age 67.90 ± 6.99	TCC=33 CG=33	TNF-α	ELISA	24 weeks, 50 minutes/session, 3 times/week	Educational control
Yang2025	Patients with mild cognitive impairment Age 31.35 ± 7.24	TCC=32 CG=30	TNF-α	Bio-Plex Pro Human Cytokine Th1/Th2 Panel	24 weeks, 1 hour/session, 3 times/week	CT
Yeh2011	Schizophrenia patients Age 67.35 ± 11.99	TCC=50 CG=50	TNF-α	NIP	12 weeks, 1 hour/session 3 times/week	Educational control
You2020	Chronic heart failure patients Age 73.86 ± 6.99	TCC=19 CG=21	IL-6, TNF-α	MMIA Multiple Magnetic Bead Immunoassay	12 weeks, 1 hour/session twice weekly	Light Physical Exercise
Yan2023	Chronic musculoskeletal pain patients Age ≥40y	TCC=25 CG=30	TNF-α	NIP	12 weeks, 1 hour/session, 3 times/week	HE
Wu2009	Diabetes patients Age 51.85 ± 6.75	TCC=20 CG=20	IL-6	ELISA	24 weeks, 1 hour/session, 3 times/week	NI
Li2013	Type 2 diabetes patients Age 57.30 ± 10.30	TCC=30 CG=30	IL-6, TNF-α	ELISA	8 weeks, 45 minutes/session, once a week	CT

TCC, Tai Chi Chuan; CG, Control group; HE, Health education; CBT, Cognitive behavioral therapy; PST, Psychosocial support therapy; NIP, No information provided; NI, No intervention; CERP, Conventional exercise rehabilitation program; CE, Conventional exercise; UC, Usual care; SST, Standard supportive therapy; CT, Conventional treatment.

*, Specific data could not be obtained subsequently.

### 3.2 Study quality and risk of bias

**Figure 2:**
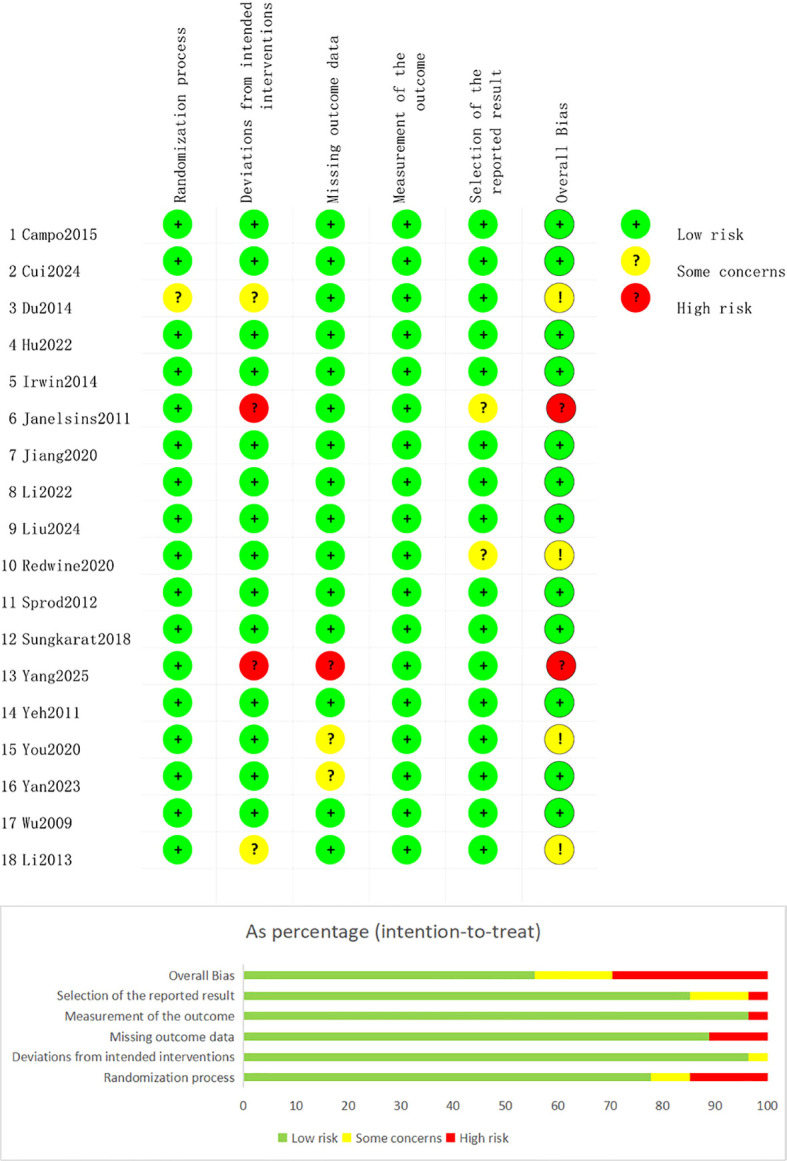
Assessment of methodological quality and risk of bias for RCTs.

An overall assessment of publication bias risk across 18 randomized controlled trials (RCTs) revealed that 12 RCTs were at low risk of bias, 2 RCTs were at high risk of bias, and 4 RCTs raised some concerns about bias. Only one study had unclear allocation of participants to treatment groups. Two studies had a high risk of bias in the section on deviation from the intended intervention, and one study had a high risk of bias in the section on missing outcome data (**Figure 2**). Two eligible quasi-experimental studies underwent quality assessment using the GRADE approach (Appendix 1).

### 3.3 Effects of TCC on pro-inflammatory gene expression

**Figure 3:**
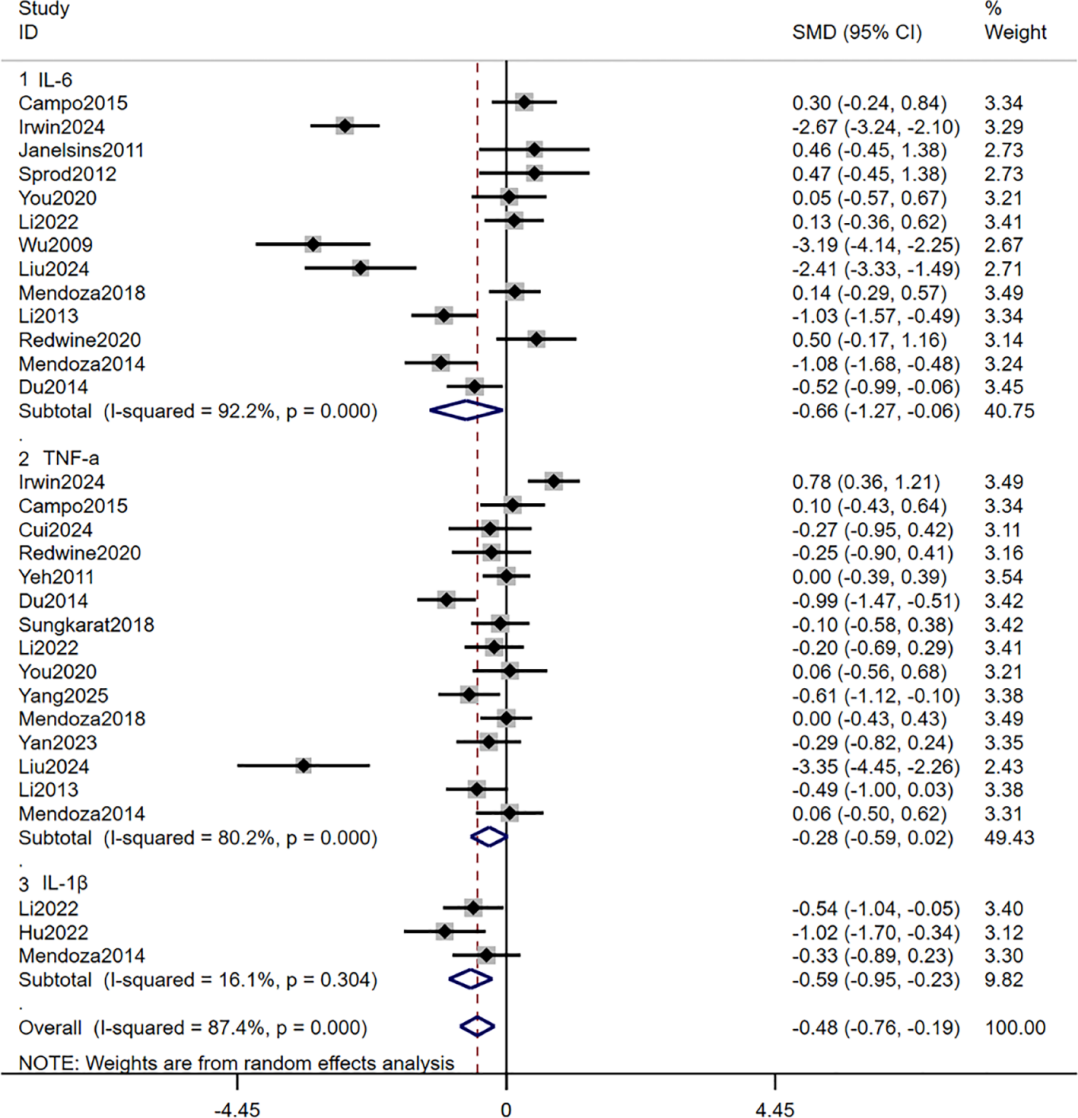
Forest plot of the effects of TCC on Pro-inflammatory Genes.

A meta-analysis of 19 studies demonstrated that TCC intervention significantly reduced the expression of pro-inflammatory genes (IL-6, TNF-α, IL-1β) (SMD = -0.48, 95% CI: -0.76 to -0.19, p < 0.01) (**Figure 3**). To further analyze the effects of TCC intervention on the NK-κB pathway, we conducted a subgroup analysis categorizing patients’ diseases according to the nine major human organ systems, providing a more intuitive reflection of TCC efficacy.

#### 3.3.1 Interleukin-6

**Figure 4:**
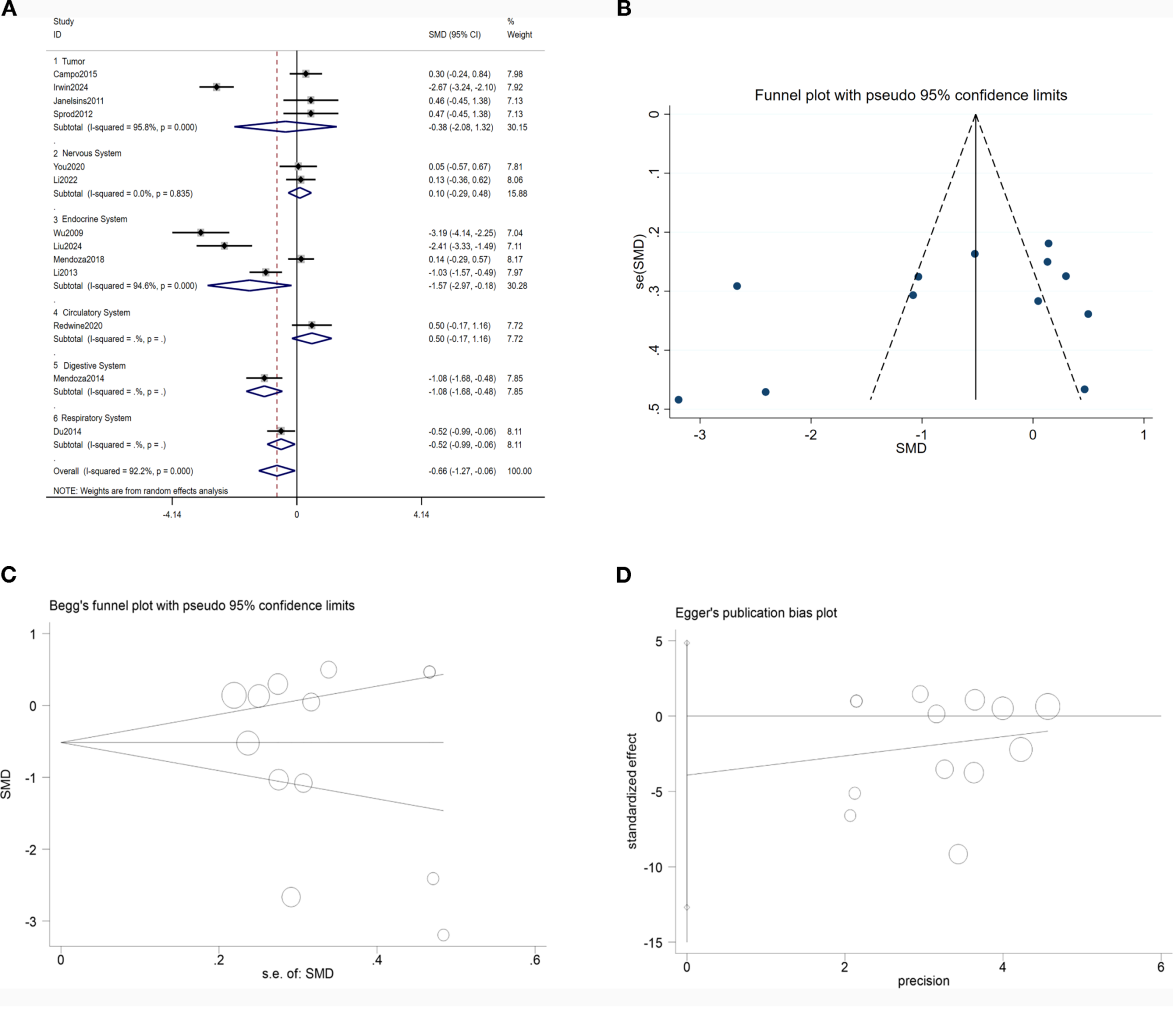
Tai Chi and IL-6. **(A)** Forest plot of the effects of TCC on IL-6; **(B)** Funnel plot; **(C)** Begg test; **(D)** Egger test.

A meta-analysis of 13 studies demonstrated that TCC significantly suppressed IL-6 proinflammatory gene expression (SMD = -0.66, 95% CI: -1.27 to -0.06, p = 0.03). Subgroup analysis revealed that TCC intervention demonstrated more pronounced effects in endocrine system disorders (diabetes, metabolic syndrome), digestive system disorders (periodontal disease), and respiratory system disorders (chronic obstructive pulmonary disease) (all P values < 0.05). Sensitivity analysis confirmed robust pooled effect sizes. Funnel plot distribution and Egger’s and Begg’s tests (p > 0.05) revealed no significant publication bias. See **Figures 4A–D**.

#### 3.3.2 Interleukin-1β

**Figure 5:**
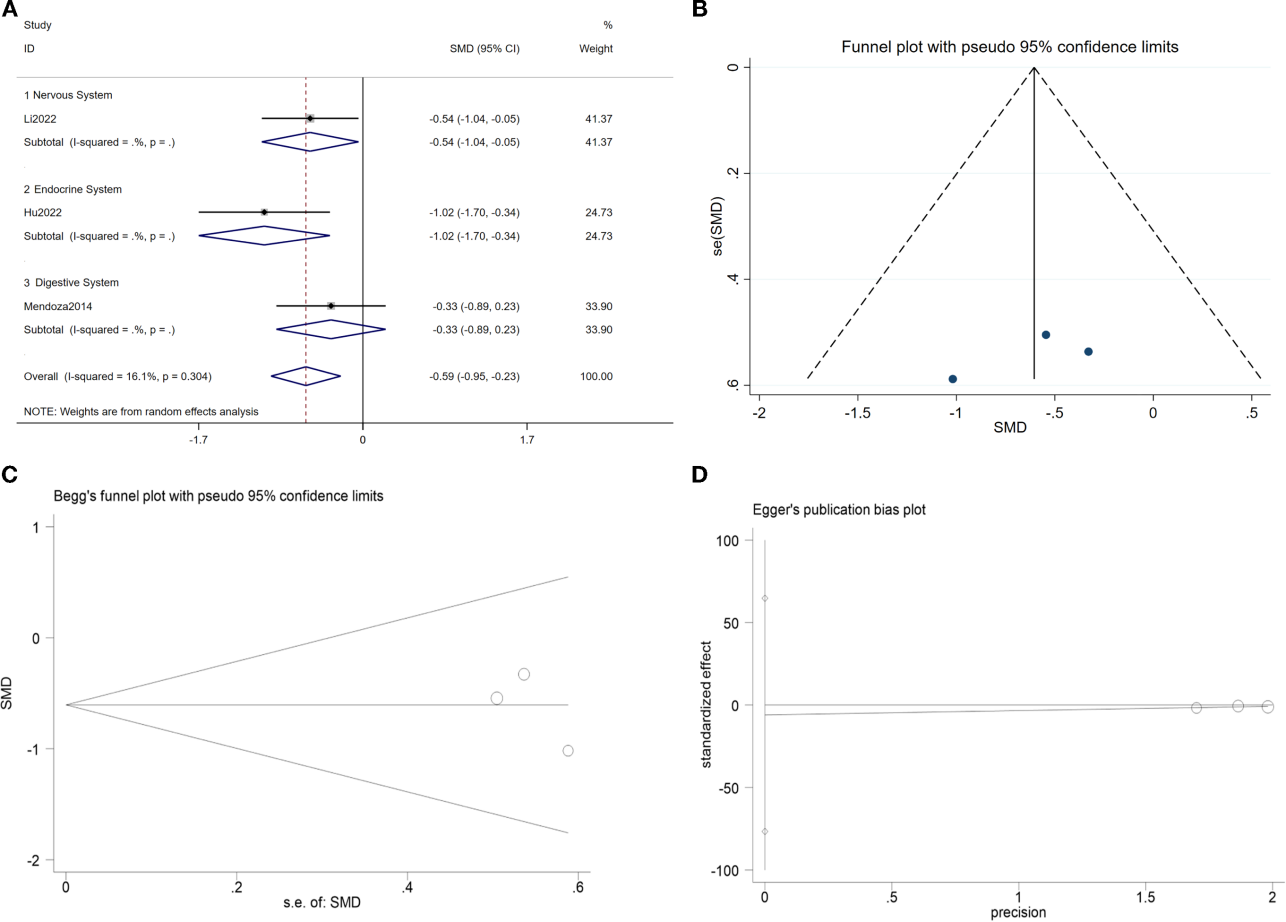
TCC and IL-1β. **(A)** Forest plot of the effects of TCC on, **(B)** Funnel plot; **(C)** Begg test; **(D)** Egger test.

A meta-analysis of three studies indicated that TCC significantly reduced IL-1β levels compared with the control group (SMD = -0.59, 95% CI: -0.95 to -0.23, p < 0.01). Notably, TCC intervention demonstrated greater efficacy in endocrine system disorders (SMD = -1.02, 95% CI: -1.70 to -0.34, p < 0.01). Neither Egger’s nor Begg’s tests detected significant publication bias (p > 0.05). See **Figures 5A–D**.

#### 3.3.3 Tumor necrosis factor-α

**Figure 6:**
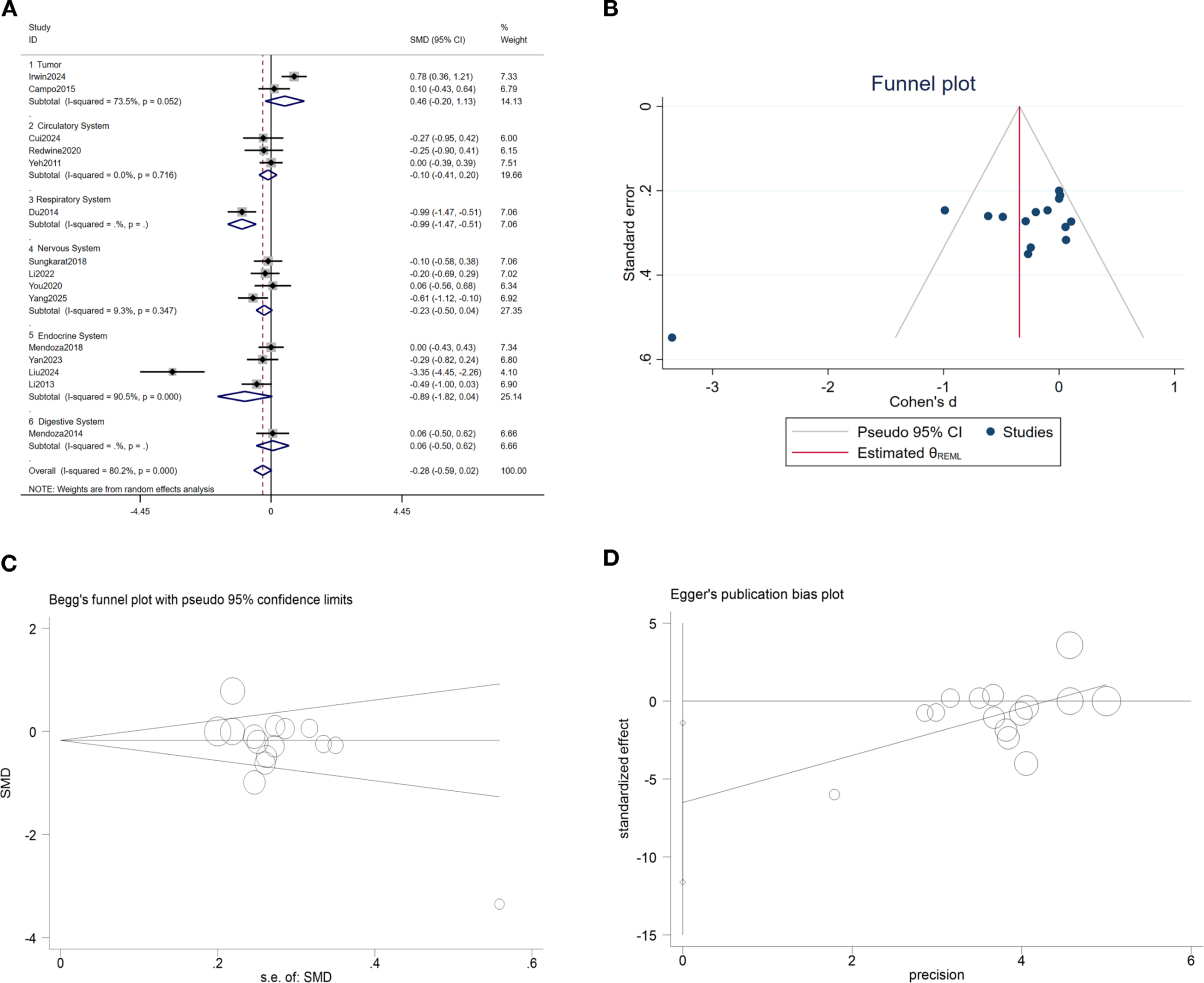
TCC and TNF-α. **(A)** Forest plot of the effects of TCC on, **(B)** Funnel plot; **(C)** Begg test; **(D)** Egger test.

A meta-analysis of 15 studies indicated that TCC had no significant effect on reducing TNF-α expression (SMD = -0.28, 95% CI: -0.59 to 0.02, p = 0.07). Subgroup analysis revealed a significant effect for respiratory diseases (chronic obstructive pulmonary disease) (SMD = -0.99, 95% CI: -1.47 to -0.51, p < 0.01). Sensitivity analyses confirmed that individual study results did not alter the overall conclusion, and the Egger test showed no abnormalities. However, the Begg test (p < 0.05) and funnel plot suggested potential publication bias. We conducted further trim-and-fill analysis, which identified no studies requiring supplementation. The combined effect size remained unchanged, indicating relatively complete evidence, though caution is warranted regarding small-sample effects. See **Figures 6A–D**.

## 4 Discussion

Previous studies have demonstrated that TCC interventions exert certain effects on inflammatory biomarkers in patients with chronic diseases, though the significance of these findings has not been entirely consistent (18, 46). To minimize the influence of heterogeneity and baseline differences, we consistently analyzed the mean change ± standard deviation from baseline to post-intervention as the outcome measure, thereby assessing the net effect of TCC interventions. To our knowledge, this is the first study to integrate the effects of TCC on the NF-κB-driven proinflammatory gene axis, selecting classic downstream target genes (IL-6, IL-1β, TNF-α) to directly reflect the intervention’s inhibitory effect on proinflammatory gene expression. To systematically elucidate the TCC intervention mechanism, we categorized diseases from included studies across nine major human systems based on modern anatomical and physiological classification standards. We then compared NF-κB axis activity across these systems under TCC intervention to identify the target system most significantly affected by Tai Chi Chuan (47). Given cancer’s anatomical characteristic of cross-system metastasis, this study uniformly classified it under the “oncology” category (48).

Our subgroup analysis revealed that TCC intervention resulted in more pronounced reductions in the expression levels of the three pro-inflammatory genes in endocrine system diseases (metabolic syndrome, diabetes), respiratory system diseases (chronic obstructive pulmonary disease), and neurological system diseases (Parkinson’s disease) compared to oncological, digestive, and circulatory system diseases. Within the NF-κB-driven pro-inflammatory gene axis, IL-6—a gene product regulated by the NF-κB signaling pathway—serves as a key STAT3 activator (3). Activated STAT3 inhibits transcription of insulin receptor substrate-1 (IRS-1) and AKT phosphorylation, impairing insulin signaling and inducing insulin resistance (49). Following TCC intervention, downstream IL-6 and TNF-α levels decrease, STAT3 activity is suppressed, insulin sensitivity increases, and consequently blood glucose levels are reduced (34). Stimuli such as PM2.5 and air pollution generate reactive oxygen species (ROS), which are the primary culprits causing oxidative damage in COPD and diabetic patients. ROS production mediates airway epithelial cell inflammation while stimulating nuclear transcription factor activation, leading to increased IL-6 transcription and expression. Neutrophil infiltration subsequently damages alveoli, resulting in impaired lung function (50). Multiple studies have demonstrated that Tai Chi Chuan possesses antioxidant properties and reduces oxidative stress (31, 44). Following TCC intervention, KEAP1-related mRNA and NRF2-associated protein levels increased, promoting up-regulation of superoxide dismutase (SOD) and down-regulation of malondialdehyde (MDA), which in turn decreased IL-6, TNF-α, and IL-1β levels.

Notably, IL-6—one of the most highly induced NF-κB-dependent cytokines—is also a dual-function factor capable of both pro-inflammatory and anti-inflammatory effects (51). Its classical signaling pathway operates independently of NF-κB (52). It binds to the IL-6 receptor (IL-6R) on the cell surface, thereby inducing gp130 homodimerization to form a high-affinity complex. Molecular interactions lead to trans-phosphorylation of Jak1 and phosphorylation of gp130 at the STAT docking site. In this process, IL-6 primarily exerts its physiological anti-inflammatory effects by promoting the expression of IL-10, IL-1 receptor antagonists, and TGF-β, while suppressing the actions of pro-inflammatory factors such as TNF-α and IL-1β.

Although IL-1β and TNF-α are downstream target genes of IL-6, they also function as upstream stimulatory signals in the NF-κB pathway, activating the IL-1R and TNFR pathways to form a positive feedback loop with NF-κB.TCC intervention can reduce pro-inflammatory factor levels by suppressing IL-1β and TNF-α expression, while also down-regulating NF-κB and inhibiting the entire NF-κB signaling pathway. This aligns with existing research findings that Tai Chi Chuan exhibits anti-inflammatory effects accompanied by “down-regulation of the NF-κB pathway” (44). Therefore, we propose that while Tai Chi Chuan suppresses IL-6 expression mediated by the NF-κB pathway, it may also significantly inhibit expression via the classical IL-6 pathway. If TCC’s suppression of TNF-α expression in the NF-κB pathway is less potent than its inhibition of the classical IL-6 pathway, pro-inflammatory factors TNF-α and IL-1β may increase, leading to enhanced NF-κB activity. When the effects on both pathways are comparable, no significant changes may be observed (39, 40). However, we propose that sustained, regular Tai Chi Chuan practice may yield greater efficacy in suppressing the NF-κB signaling pathway than the IL-6 classical pathway. This is because activation of the sympathetic nervous system signals immune cells via β-adrenergic receptors, leading to NF-κB activation and subsequent up-regulation of IL-6, TNF-α, and IL-1β levels (53). Existing research has demonstrated that Tai Chi Chuan effectively promotes reduced sympathetic nervous system activity (54), thereby decreasing NF-κB activation.

Due to the limited number of standardized randomized controlled trials, this study has the following limitations. First, the 20 included studies varied in their Tai Chi intervention protocols, such as the type, frequency, duration per session, and total duration (**Table 1**). To our knowledge, Tai Chi encompasses multiple styles, including Yang-style, Wu-style (Hao-style), Chen-style, Wu-style (Yi-style) and the internationally recognized 24-form Tai Chi (standard Tai Chi). Each style features distinct movements and forms, with varying intensity levels. Research indicates that Yang-style Tai Chi is classified as a moderate-intensity activity, while the 24-form Tai Chi is lower in intensity compared to traditional styles (55). This implies that when total training duration is low or intervention protocols differ, it may affect the significance and reliability of the aforementioned pathway expression results. This also places higher demands on future experiments. Second, some included studies measured multiple pro-inflammatory genes simultaneously rather than tracking changes in a single, identical biomarker per study, potentially introducing heterogeneity and non-significant effects. Third, not all included studies explicitly calculated sample sizes, potentially resulting in insufficient statistical power. This may compromise the reliability of the findings in this study. For example, despite applying trimming methods to our TNF-α studies, there remains a possibility of bias arising from small sample sizes.

Although downstream target gene analysis revealed Tai Chi Chuan’s impact on the NF-κB-driven pro-inflammatory gene axis, direct evidence for pathway-wide expression remains lacking—specifically, detection of core pathway components such as NF-κB, p65 (RelA), IκBα, and IKKβ. Few randomized controlled trials have measured these factors post-Tai Chi Chuan intervention. Future studies should conduct more randomized controlled trials, incorporate peripheral blood mononuclear cell sampling, and use ELISA or Western blot to detect phosphorylation levels of NF-κB p65, IκBα, and IKKβ. This would provide a concise, reproducible method to directly validate Tai Chi Chuan’s regulatory effects on this pathway. Furthermore, whether Tai Chi Chuan involves the production and release of specific bioactive substances during intervention to participate in pathway inhibition requires extensive experimentation and further investigation.

## 5 Conclusion

This systematic review and meta-analysis included 20 randomized controlled trials, using IL-6, TNF-α, and IL-1β as downstream markers, demonstrating that Tai Chi Chuan intervention significantly inhibits the NF-κB signaling pathway in adult patients with chronic diseases. The most pronounced benefits were observed in endocrine, respiratory, and neurological disorders. Mechanistically, Tai Chi Chuan achieves this through two pathways: first, by reducing sympathetic nervous system excitation to inhibit NF-κB activity, thereby decreasing expression of downstream pro-inflammatory genes. Second, direct down-regulation of IL-6, TNF-α, and IL-1β disrupt the positive feedback loop of the pathway, and inhibition of IL-6 is accompanied by inhibition of its classical pathway. Suppression of pro-inflammatory gene expression implies normalization of the immune system activity long activated by pro-inflammatory gene expression, which in turn slows down the inflammatory destruction of the endocrine, respiratory, and cardiovascular systems by systemic chronic inflammation, which is directly manifested in the remission of the chronic disease course. Further research is needed to determine the persistence of Tai Chi Chuan’s anti-inflammatory mechanisms, the stability of its therapeutic effects, the optimal conditions for implementation, and whether its influence on molecular pathways exhibits stable specificity.

## Data Availability

The raw data supporting the conclusions of this article will be made available by the authors, without undue reservation.
